# Effects of bed nets and anti-malaria drugs use on childhood mortality in Kenya’s malaria endemic and epidemic areas

**DOI:** 10.1186/s12889-015-1398-x

**Published:** 2015-01-29

**Authors:** Boniface O K’Oyugi

**Affiliations:** Population Studies and Research Institute, University of Nairobi, P.O. Box 30197, 00100 Nairobi, Kenya

**Keywords:** Childhood mortality, Bed nets, Anti-malaria drugs, Malaria epidemic and endemic, Kenya

## Abstract

**Background:**

The Kenya Demographic and Health Surveys (KDHS) data collected since 1989 indicate that malaria prone areas have consistently recorded the highest childhood mortality rates. Malaria control programme information also indicates that malaria contributes to about 20 per cent of the deaths among under-five year old children. The 2009–2017 National Malaria Strategy is being implemented to reduce malaria morbidity and mortality. Its key interventions include: bed nets use; anti-malaria drugs use during pregnancy for prevention; and, prompt treatment using anti-malaria drugs of children with fever. This study seeks to establish differentials in childhood mortality rates by these interventions in three malaria prone areas defined as highland epidemic, coast endemic and lake endemic. It also seeks to determine the effects of these interventions on childhood mortality.

**Methods:**

The data used is drawn from the 2008/9 KDHS. The study sample consists of 3,728 children born less than 60 months prior to the survey. The direct demographic method for estimation of childhood mortality rates and multivariate Poisson regression models are used to analyse the data.

**Results:**

The findings show that use of bed nets and anti-malaria drugs are not high in Kenya’s malaria prone areas. On the average, only about 60% of the children are found to be in higher use category for each of the three intervention measures. The childhood mortality rates show that higher use of prompt treatment with anti-malaria drugs for children with fever has lower infant and under-five mortality rates in all malaria epidemic and endemic areas compared to lower use. Higher bed nets use has lower childhood mortality rates compared to lower use in coast and lake endemic areas. Higher use of anti-malaria drugs during pregnancy for prevention has lower childhood mortality rates in highland epidemic and lake endemic areas compared to lower use. The regression models fitted show that in highland epidemic area, higher use of anti-malaria drugs during pregnancy for prevention has significant reduction effect on childhood mortality compared to lower use in the presence of breastfeeding duration, toilet facility type and age of child variables. The regression models also show that in combined malaria prone areas, higher prompt treatment of children with fever using anti-malaria drugs has significant reduction effect on both infant and child mortality compared to lower prompt treatment in the presence of breastfeeding duration and toilet facility type variables.

**Conclusion:**

This study underscore the need for increasing uptake of malaria interventions and complementing them with longer breastfeeding duration and improved toilet facility in efforts towards reducing infant and child mortality rates in Kenya’s malaria prone areas. There is also need to improve quality of individual household data for malaria module in future KDHS undertakings.

## Background

In Kenya, malaria accounts for about 20% of childhood mortality [1]. The country is classified into five zones or areas according to malaria transmission intensity and seasonality [2]. These are: highland epidemic area; lake endemic area; coast endemic, semi-arid and seasonal areas; and, low risk area. Kenya conducts national demographic and health surveys every five years since 1989 as part of the world-wide Demographic and Health Surveys (DHS) programme. All the data from these past surveys have shown that coast and lake endemic malaria areas have the highest infant and child mortality rates in Kenya [3]. To address the malaria problem, the country is implementing the 2009–2017 National Malaria Strategy [4] as part of the health sector programmes within the framework of the Kenya Vision 2030 long term development blue print. The strategy has four priority interventions: insecticide treated mosquito nets; indoor residual spraying; intermittent preventive treatment of pregnant women with sulfadoxine-pyrimethemic; and, diagnosis with rapid diagnostic tests or microscopy and treatment with artemisinin based combination therapy. This strategy is complementing the integrated management of childhood illnesses programme which is also being implemented as part of the global initiative for improving child survival.

Under-five mortality rate in Kenya recently declined from 115 per 1000 live births in 2003 to 74 per 1,000 in 2008/9 and there are indications that the reduction can partly be attributed to the impact of malaria intervention measures [3]. The main objective of this study is to determine the effects of the priority interventions measures on childhood mortality in malaria endemic and epidemic areas. The 2008/9 Kenya Demographic and Health Survey (KDHS) data has no information on indoor residual spraying hence this study focuses on only three intervention measures: mosquito bed nets; treatment of pregnant women with anti-malaria drugs for prevention; and prompt treatment of children with fever using anti-malaria drugs. The malaria prone areas considered are highland epidemic, coast endemic and lake endemic areas.

This study has two specific objectives. First, is to determine whether or not childhood mortality variations exist by intervention measure and malaria prone area. It should be noted that infant mortality rate and under-five mortality rate are two of the standard childhood mortality indicators for reporting progress towards attainment of Millennium Development Goal (MDG) number 5. Assessments of childhood mortality levels and patterns by malaria prone area and intervention measure are done using the two standard indicators. The second specific objective is to determine the effects of these malaria interventions on childhood mortality in each and all malaria prone areas taking into account other factors with significant impact on childhood mortality.

Information on past studies focusing on malaria interventions and childhood mortality, done as separate topics, are readily available. However, very scanty information exists on the impact of malaria interventions and childhood mortality. Past review of studies on malaria and childhood mortality inter-linkages concluded that malaria do: give rise to high fever and anaemia; obstruct the blood vessels of the brain resulting in coma and death; contribute to complex malnutrition and infection increasing in the risk of child death; and, retard intra-uterine growth of foetus resulting in low birth weights in new born infants thus indirectly increasing the risk of neonatal mortality [5]. Hospital admission data of infants across four sites including Kenya show that there is significant clinical protection against severe malaria morbidity in the first three months of life [6]. This protection appears to be derived from Immunoglobulin G transferred through the placenta [7,8]. Severe life threatening malaria in infancy usually occurs during later part of the first year of life [9-11].

Some of the studies done in Kenya and sub-Sahara Africa on impact of malaria interventions on childhood mortality have concluded as follows: (i) insecticide spraying campaigns in Kenya, Nigeria and Tanzania in the 1950s and 1970s reduced mortality among infants and children aged 1–4 years by as much as 40-50% [12]; (ii) malaria chemoprophylaxis and bed nets reduced under-five mortality by 42% [13]; (iii) rapid intensification of malaria control in Rwanda and Ethiopia among children under-five focusing on bed nets and ACT reduced malaria deaths by 62 and 67% in Ethiopia and Rwanda, respectively [14]; rapid scale-up of malaria control in Zanzibar reduced malaria deaths in health facilities by 90% [15]; and, (iv) malaria admission rates from coastal Kenya fell dramatically up to 90% in some hospitals [16-18].

Studies undertaken to determine the contribution of malaria interventions on the decline of Kenya’s infant mortality rate from 77 per 1,000 live births in 2003 to 52 per 1,000 in 2008/9 have provided varying estimates ranging from 33 to 58% [3,19,20]. A more recent study carried out by Pathania in 2014 concluded that: prior to intervention, infant mortality in malaria regions was substantially higher than in non-malaria regions due to higher post-neonatal mortality in malaria regions; after intervention, there was significant fall in post-neonatal mortality in malaria regions compared to non-malaria regions; renewed malaria campaign led to fall in post-neonatal mortality by 22 to 25% per 1,000 live births in malaria regions; and, differentials in infant mortality in malaria regions were being driven by intensity of malaria control and relative improvements in socio-economic conditions [19].

### Data

The data used in this study is from the 2008/9 KDHS [3]. This was a national survey carried out by the Kenya National Bureau of Statistics (KNBS) as part of the world-wide DHS programme. The woman’s questionnaire implemented covered 13 topics. The topic on immunization, nutrition and childhood illnesses included information on malaria. The focus of this study is children born less than 60 months prior to the survey to women in malaria prone areas. To subset malaria prone areas data from other areas in the KDHS data, the study has used survey district codes obtained from the 2010 Kenya Malaria Indicator Survey (KMIS) [2]. The 2008/9 KDHS and 2010 KMIS were conducted using the same national sampling frame hence shares same district codes. Although previous KDHS data had malaria module, the information has not used due to differences in their sampling frames.

The percentage distribution of the children in the malaria prone areas is given on Table 1. The study sample has 3,278 children. The variable on use of anti-malaria drugs for prompt treatment of children with fever has the highest percentage of missing values (10% overall and 53% in highland epidemic area) but it is retained in the analysis due to its importance to this study. All the other variables have very low missing values (2% or less).

**Table 1:** **Percentage distribution of children born less than 60 months prior to 2008/9 KDHS in Kenya’s malaria prone areas**

**Variable**	**Malaria prone area**			
	**Highland epidemic**	**Coast endemic**	**Lake endemic**	**All**
Bed nets use by children				
Higher (>60%)	26.3	82.2	64.9	57.9
Lower (<= 60%)	73.7	17.8	35.1	42.1
Number of children	938	770	1,570	3,278
Prompt treatment with anti-malaria drugs for children with fever				
Higher (>45%)	73.3	57.9	54.5	58.7
Lower (<= 45%)	26.7	42.1	45.5	41.3
Number of children	445	770	1,407	2,622
Anti-malaria drugs use during pregnancy for prevention				
Higher (>44%)	44.5	57.7	70.9	60.2
Lower (<= 44%)	55.5	42.3	29.1	39.8
Number of children	938	770	1,570	3,278
Mean breastfeeding duration in months				
Longer (>13.5)	86.1	52.5	47.6	59.8
Shorter (<= 13.5)	13.9	47.5	52.4	40.2
Number of children	938	770	1,570	3,278
Toilet facility type				
Improved	13.4	34.1	16.1	19.6
Non improved	86.6	65.9	83.9	80.4
Number of children	933	756	1,548	3,237
Age of child				
12-59 months	87.5	85.5	85.7	86.2
0 – 11 months	12.5	14.5	14.3	13.8
Number of children	938	770	1,570	3,278

Note: Cut point values for the categorical variables are discussed in section on Methods.

## Methods

This study uses two main methods of data analysis: direct demographic estimation method and multivariate Poisson regression. Direct demographic method for estimation of childhood mortality rates is used to compute life table probabilities of children dying before attaining age 1 year (infant mortality rate) and age 5 years (under-five mortality rate). The procedure is described in detail in the manual on Tools for Demographic Estimation [21]. In this study, the mortality rates computation process entails five steps. First, is to segment childhood ages into two (0–11 months and 12–59 months). Second, is to calculate total number of dead children and total months of exposures in each of the two age segments using information on age at death in months for the dead children and age in months at the time of survey for the living children. Third, is to calculate risk of death in each age segment by dividing the total dead children by total months of exposures. Fourth, is to convert the risks of death in the age segments into life table survival probabilities from birth to age 1 year and age 5 years. The final step is to derive life table probabilities of dying before 1 year and 5 years from the life table survival probabilities. The Statistical Package for Social Sciences (SPSS) computer software is used to generate input data for the computations of infant and under-five mortality rates.

The multivariate Poisson regression analysis is applied to determine the effects of the malaria intervention variables on childhood mortality rates. This regression method is able to provide rate ratios of covariates in the analysis and also takes care of violation of the assumption on equal mean and variance at each level of covariate. The assumption violation is common when dealing with analysis of rare events such as deaths. The Poisson regression model specifications and its applications are described in detailed by Lawless [22]. Its application is also suitable for count data designed to estimate rate of occurrence using counts, exposures and a set of explanatory or predictor variables. The mathematical Equation 1 represents the Poisson regression model in log linear format.

1 $$ \ln (Y)- \ln (T) = \alpha + \boldsymbol{\beta} i\boldsymbol{X} i $$

Where:-

Y is count of occurrences

T is total exposures

*α* is the intercept term

*Xi* is a vector of regression variables

*βi* is a vector of regression coefficients

The dependent variable in this study is the natural logarithm of total number of dead children in age segment. The off-set variable is the natural logarithm of total months of exposures in age segment. The explanatory variables are malaria interventions and other variables serving as controls in the analysis. Malaria intervention variables are bed nets use by children, prompt treatment with anti-malaria drugs for children with fever and anti-malaria drugs use during pregnancy for prevention. Initially, a total of 17 possible explanatory variables were considered based on results of recent studies on determinants of child mortality in Kenya [5,23]. In this analysis, only 4 are found to be statistically significant based on bivariate regression models fitted. These are mean breastfeeding duration, toilet facility type, age segment of the child and type of birth (single/multiple). However, type of birth is excluded due to rare occurrence of multiple births that cannot allow fitting of regression models involving the variable for each malaria prone area.

The 3 malaria intervention variables are computed from information on: (i) reported bed nets use on night preceding the survey for surviving children; (ii) reported prompt treatment with anti-malaria drugs for surviving children who had fever within two weeks prior to the survey; and, (iii) reported anti-malaria drugs use during last pregnancy for prevention among individual mothers of the living and dead children. The 3 variables used as controls in the models are computed from information on: (i) reported breastfeeding durations of children belonging to last closed birth intervals; (ii) reported type of household toilet facility; and, (iii) reported age at death in months for individual dead children and age in months during the survey for individual living children.

The procedure used to compute all the community level variables in the analysis require some comments. The survey districts codes are applied to obtain aggregate district mean values for each individual variable. The district mean values for each variable are arranged in descending order. The 60 and 40 percentages rule is applied to classify the values in each variable distribution into 2 categories as higher and lower, respectively. The cut points for the categorical community level variables in the analysis are as follows: (i) bed nets use by children (less or equal to 60% as lower and over 60% as higher); (ii) prompt anti-malaria treatment of child with fever (less or equal to 45% as lower and over 45% as higher); (iii) anti-malaria drug use during pregnancy for prevention (less or equal to 44% as lower and over 44% as higher); and, (iv) mean breastfeeding duration (13.5 months or lower as shorter and over 13.5% as longer).

Fitting of the regression models is also guided by the 1984 Mosley-Chen analytical framework for child survival [24]. The basic assumption in the framework is that child mortality is a result of background socio-economic variables operating through proximate determinants or by proximate determinants directly. The framework allow use of contextual/community variables. In this study, the 3 malaria intervention variables and mean breastfeeding duration variable are treated as socio-economic contextual/community variables. Type of toilet facility and age of the child variables are treated as proximate determinants variables. The two proximate variables are computed using information on household amenities and individual child, respectively.

The multivariate Poisson regression models are fitted for separate and all malaria areas. The regression models are classified into sub models and full models. The sub models fitted contain only the malaria intervention variables. This is to facilitate assessment of the malaria intervention variables on childhood mortality without controlling for effects of other variables. The full models fitted contain the malaria intervention variables and the three variables used in this study as controls. This is to allow assessment of relative effects of the malaria intervention variables in the presence of the variables used as controls in the study.

The rate ratio of a variable reference category is one (1.000). Rate ratios greater than 1.000 indicate increased likelihood of childhood mortality while rate ratios less than 1.000 indicate reduced likelihood of mortality relative to the reference category. In this analysis, only rate ratios that are statistically significant at least at 5% level of significance are reported as having significant effect. The study uses the Generalized Estimation Equations subroutine in the SPSS computer software to fit the multivariate Poisson regression models.

Separate analysis is also done to determine the effects of these malaria intervention variables on infant mortality (age segment 1–11 months) and child mortality (age segment 12–59 months). This strategy is informed by the finding in literature review to the effect that malaria is more severe from the later periods of infancy [9-11].

## Results

### Use of bed nets and anti-malaria drugs

The findings on usage of bed nets and anti-malaria drugs are already presented on Table 1. On bed nets, the results show that coast endemic area has the highest use (82% in higher category) and highland epidemic area has the lowest (26% in higher category). On prompt treatment with anti-malaria drugs for children with fever, the highland epidemic area has the highest (73% in higher category) and lake endemic area has the lowest (55% in higher category). On anti-malaria drugs use during pregnancy for prevention, lake endemic area has the highest (71% in the higher category) and highland epidemic has the lowest (45%). On the overall, the highland epidemic area has the lowest usage rate for the combined 3 interventions when compared to the other areas since it is ranked lowest in 2 out of the 3 interventions. The area has also the highest missing values for the intervention variable on prompt treatment (52%).

### Infant and under-five mortality differentials by malaria intervention measures

The results on infant and under-five mortality rates obtained using direct demographic technique are presented on Table 2. On bed nets use intervention, the findings show that children in higher category have lower infant and under-five mortality rates in two malaria endemic areas (lake and coast) compared to their counterparts in lower category. The reverse is the case in highland and combined malaria prone areas. On prompt use of anti-malaria drugs for treatment of children with fever, the results show that children in the higher category have lower infant and under-five mortality rates in all the three malaria prone areas when compared to their other counterparts. On use of anti-malaria drugs during pregnancy for prevention, the results show that children in the higher category have lower infant and under-five mortality rates when compared to lower use in highland epidemic and lake endemic areas. However, the reverse situation is the case in coast endemic and for under-five mortality in combined malaria areas.

**Table 2:** **Infant and under-five mortality rates for malaria intervention measures in Kenya’s malaria prone areas**

**Intervention measure**	**Highland epidemic**		**Coast endemic**		**Lake endemic**		**All**	
	**Mortality rates per thousand live births**							
	**IMR**	**U5MR**	**IMR**	**U5MR**	**IMR**	**U5MR**	**IMR**	**U5MR**
Bed nets use by children								
Higher (>60%)	47	104	51	62	64	118	58	97
Lower (<= 60%)	24	36	67	67	77	126	49	75
Prompt treatment with anti-malaria drugs for children with fever								
Higher (>45%)	16	34	44	49	63	101	55	79
Lower (<= 45%)	34	72	62	73	72	141	60	103
Anti-malaria drugs use during pregnancy for prevention								
Higher (>44%)	36	72	67	79	63	112	55	87
Lower (<= 44%)	43	88	38	42	82	144	52	89
Malaria prone area	30	53	54	63	69	121	54	88

Key: IMR is Infant Mortality Rate; and, U5MR is Under-five Mortality Rate.

Note: Cut point values for the categorical variables are discussed in section on Methods.

### Effects of bed nets and anti-malaria drugs use on childhood mortality

Childhood mortality covariates rate ratios obtained from Poisson regression models fitted for individual and combined malaria areas are presented on Table 3 for both sub and full models. The sub models have only the 3 malaria intervention variables while the full models have 3 additional variables used as controls in this study. These additional variables are mean breastfeeding duration, toilet facility type and age segment of child. The findings are presented for each malaria prone area.

**Table 3:** **Poisson regression models rate ratios of under-five mortality covariates for Kenya’s malaria prone areas**

**Covariate**	**Highland epidemic**		**Coast endemic**		**Lake endemic**		**All malaria prone**	
	**Sub model**	**Full model**	**Sub model**	**Full model**	**Sub model**	**Full model**	**Sub model**	**Full model**
Bed nets use by children								
Higher (>60%)	3.478^***^	1.961	0.631	0.449	0.502	1.340	1.654^**^	1.179
Lower (<= 60%) [RC]	1.000	1.000	1.000	1.000	1.000	1.000	1.000	1.000
Prompt treatment with anti-malaria drugs for children with fever								
Higher (>45%)	0.516	0.877	1.588	7.217^**^	0.704	0.984	0.610^**^	0.896
Lower (<= 45%) [RC]	1.000	1.000	1.000	1.000	1.000	1.000	1.000	1.000
Anti-malaria drugs use during pregnancy for prevention								
Higher (>44%)	0.608^*^	0.344^**^	1.488	0.658	0.635	0.710	1.321	0.950
Lower (<= 44%) [RC]	1.000	1.000	1.000	1.000	1.000	1.000	1.000	1.000
Mean breastfeeding duration in months								
Longer (>13.5)	-	0.412	-	1.414	-	0.389^***^	-	0.424^***^
Shorter (<= 13.5) [RC]	**-**	1.000	-	1.000	-	1.000	-	1.000
Toilet facility type								
Improved	-	1.503	-	0.320^***^	-	0.865	-	0.635
Non improved [RC]	-	1.000	-	1.000	-	1.000	-	1.000
Age of child in months								
12-59	-	0.002^***^	-	0.002^***^	-	0.014^***^	-	0.008^***^
0 – 11 [RC]	-	1.000	-	1.000	-	1.000	-	1.000

Notes:

1. RC – Reference category; ***p < 0.000; **p < 0.01; *p < 0.05.

2. Cut point values for the categorical variables are discussed in section on Methods.

#### Highland epidemic area

In highland epidemic area, the sub model results show that higher use of anti-malaria drugs during pregnancy for prevention has significant reduction effect on childhood mortality compared to lower use. The sub model results also show that higher use of bed nets has significant effect on childhood mortality increase compared to lower use.

The full model results show that higher use of anti-malaria drugs during pregnancy for prevention has statistically significant reduction effect on childhood mortality relative to those with lower use.

#### Coast endemic area

In coast endemic area, the sub model results reveal that none of the malaria intervention variables have significant effect on childhood mortality. On the other hand, the full model show that higher prompt treatment using anti-malaria drugs for children with fever has significant reduction effect on childhood mortality when compared to lower prompt treatment.

#### Lake endemic area

In lake endemic area, the sub model and full model results show that none of the malaria intervention variables has significant effect on childhood mortality.

#### All malaria prone areas

In all malaria prone areas combined, the sub model results show that higher prompt treatment using anti-malaria drugs for children with fever has significant reduction effect on childhood mortality compared to lower prompt treatment. The sub model also show that, higher bed nets use has significant increase on childhood mortality compared to lower use.

The full model results reveal that none of the malaria intervention variables has significant effect on childhood mortality.

### Effects of bed nets and anti-malaria drugs use on infant and child mortality

Further scrutiny of under-five mortality covariates presented on Table 3 reveal that the variable age segment has significant effect on childhood mortality in individual and the combined malaria prone areas. Covariates rate ratios for regression models fitted to determine the effects of the intervention variables and 2 additional variables (mean breastfeeding duration and toilet facility type) used as controls in the models are presented on Table 4 for infant mortality (age segment 1–11 months) and child mortality (age segment 12–59 months). The study findings are presented for each age segment.

**Table 4:** **Poisson regression models rate ratios of infant and child mortality covariates for Kenya’s malaria prone areas**

**Covariate**	**0-11 months**		**12-59 months**	
	**Sub model**	**Full model**	**Sub model**	**Full model**
Bed nets use by children				
Higher (>60%)	1.171	1.259	1.504	1.255
Lower (<= 60%) [RC]	1.000	1.000	1.000	1.000
Prompt treatment with anti-malaria drugs for children with fever				
Higher (>45%)	0.819	0.763^***^	0.547^***^	0.620^**^
Lower (<= 45%) [RC]	1.000	1.000	1.000	1.000
Anti-malaria drugs use during pregnancy for prevention				
Higher (>44%)	0.941	0.959	1.655	1.275
Lower (<= 44%) [RC]	1.000	1.000	1.000	1.000
Mean breastfeeding duration				
Longer (>13.5 months)	-	0.481^***^	-	0.422^*^
Shorter (<= 13.5 months) [RC]	**-**	1.000	-	1.000
Toilet facility type				
Improved	-	0.295^***^	-	0.436^***^
Non improved [RC]	**-**	1.000	**-**	1.000

Notes:

1. RC – Reference category; ***p < 0.000; **p < 0.01; *p < 0.05.

2. Cut point values for the categorical variables are discussed in section on Methods.

#### Infant mortality

The sub model results reveal that none of the malaria intervention variables has significant effect on infant mortality. The full model results show that higher prompt treatment using anti-malaria drugs for children with fever has significant reduction effect on infant mortality compared to lower prompt treatment. The model also show that mean breastfeeding duration and toilet facility type variables included in the analysis as controls have significant reduction effect on infant mortality.

#### Child mortality

The sub-model results show that higher prompt treatment using anti-malaria drugs for children with fever has significant reduction effect on child mortality compared to lower prompt treatment. The full model results also show that higher prompt treatment has significant reduction effect on child mortality compared to lower prompt treatment. It shows further that mean breastfeeding duration and toilet facility type variables included in the analysis as controls have significant reduction effect on child mortality.

## Discussion and conclusion

The study findings on percentage distributions of bed nets and anti-malaria drugs use indicate that the uptake of these interventions is not high in malaria prone areas. This is demonstrated by the low cut point values for higher categories of malaria intervention variables (60%, 45% and 44% respectively for bed nets, prompt treatment of children with fever and anti-malaria drugs during pregnancy for prevention). Data on prompt treatment of children with anti-malaria drugs for children with fever is generally poor. The highland epidemic area has the highest missing values (52%) for this particular variable.

The infant and under-five mortality rates for the 3 malaria intervention variables, presented on Table 3, do not conform to the expected pattern in all malaria prone areas except for prompt treatment with anti-malaria for children with fever. Higher use category for each malaria intervention variable was expected to have lower mortality rate compared to lower use category in each malaria prone area. This study finding can be interpreted to mean that the intervention variables depicting conformity in a number of areas are just giving indicative association of higher usage with lower mortality rates.

The rate ratios of Poisson regression models fitted for each and combined malaria prone areas given on Table 3 show that in highland epidemic area, higher use of anti-malaria drugs during pregnancy for prevention has significant reduction effect on childhood mortality relative to lower use in the presence of the 3 variables included in the full models as controls. This can be interpreted to mean that in highland epidemic area, use of anti-malaria drugs during pregnancy for prevention is an important intervention measure for childhood mortality reduction in the presence of the 3 variables included in the full model. The rate ratios for the combined malaria prone areas given on Table 4 show that in malaria prone areas higher prompt treatment with anti-malaria drugs for children with fever has significant reduction effect on both infant and child mortality rates compared to lower prompt treatment. This result do re-affirm the findings of the mortality rates presented on Table 2 which also showed higher that prompt treatment with anti-malaria drugs for children with fever has lower infant and child mortality rates in each malaria prone area compared to lower prompt treatment. The findings also demonstrate the reduction effects of longer breastfeeding durations and improved toilet facility type on infant and child mortality in malaria prone areas.

The study results have indicated that in malaria prone areas, the uptake of malaria interventions is not as high as expected as reflected by the usage percentages for three interventions analysed. Programme efforts should be directed towards increasing their uptake to a near universal level (100%) so as to contribute to attainment of the national target for MDG 5 on infant and child mortality rates since malaria endemic areas have also the highest childhood mortality in Kenya.

The results have also shown significant reduction effect of higher prompt treatment with anti-malaria drugs for children with fever on both infant and child mortality. Child health care services provided in the health facilities and communities should be enhanced to promote uptake of the malaria intervention programmes. The malaria interventions should also be complemented with breastfeeding and sanitation promotion programmes to accelerate the pace of infant and child mortality reduction in Kenya’s malaria prone areas.

The major limitation of the data used and by extension the study is the fact that community level behaviour may not necessarily reflect individual household behaviour especially on health care seeking. This study adopted the strategy of computing community level variables to deal with the problem of large missing values in individual household malaria data collected in the 2008/9 KDHS. Since KDHS data complements periodic KMIS data and it is also rich in variables useful for further analysis of health and demographic outcomes, future KDHS undertakings should strive to improve the quality of malaria information collected in the households. There is also need for separate analysis of infant and child mortality when assessing the effects of malaria intervention measures on childhood mortality.

### Ethical approval

The study used a copy of the KDHS 2008–09 data file obtained from Kenya National Bureau of Statistics. No ethical approval was needed since this data file had all household and cluster codes already scrambled to protect identities of individual respondents so as to maintain confidentiality of the HIV/AIDS information contained in it. The research committee of the Kenya Medical Research Institute had given ethical cleared for the KDHS 2008–09.

## References

[1] Division of Malaria Control (2011). Malaria Fact Sheet.

[2] Division of Malaria Control (2011). 2010 Kenya MALARIA Indicator Survey.

[3] Kenya National Bureau of Statistics (KNBS) and ICF Macro (2010). Kenya Demographic and Health Survey 2008-09.

[4] Division of Malaria Control (2009). National Malaria Strategy 2009-2017.

[5] K’Oyugi BO (1992). The Impact of Household and Community Level Environmental Factors on Infant and Child Mortality in Rural Kenya.

[6] Snow RW, Nahlen B, Palmer A, Donnelly CA, Gupta S, Marsh K (1998). Risk of severe malaria among African infants: direct evidence of clinical protection during early infancy. J Infect Dis.

[7] Amaratunga C, Lopera-Mesa TM, Brittain NJ, Cholera R, Arie T, Fujioka H (2011). A role for foetal haemoglobin and maternal immune IgG in infant resistance to Plasmodium falciparum malaria. PLoS One.

[8] McGregor IA (1986). The development and maintenance of immunity to malaria in highly endemic arrears. Cli Trop Med Commun Dis.

[9] Colbourne MJ, Edington GM (1954). Mortality from malaria in Accra. J Trop Med Hyg.

[10] Garnham PCC (1949). Malaria immunity in Africans: effects in infancy and early childhood. Ann Trop Med Parasitol.

[11] Macdonald G (1950). The analysis of malaria parasite rates in infants. Trop Dis Bull.

[12] Payne D, Grab B, Fontaine RE, Hempel JHG (1976). Impact of control measures on malaria transmission and general mortality. Bull World Health Organ.

[13] Alonso PL, Lindsay SW, Armstrong JRM, de Francisco A, Shenton FC, Greenwood BM (1991). The effect of insecticide-treated bed nets on mortality of Gambian children. Lancet.

[14] Otten M, Aregawi M, Were W, Karema C, Medin A, Korenromp W (2009). Initial evidence of reduction of malaria cases and deaths in Rwanda and Ethiopia due to rapid scale-up of malaria prevention and treatment. Malar J.

[15] Aregawi MW, Ali AS, Al-mafazy A, Molteni F, Katikiti S, Warsame M (2011). Reductions in malaria and anaemia case and death burden at hospitals following scale-up of malaria control in Zanzibar, 1999-2008. Malar J.

[16] Okiro EA, Hay SI, Gikandi PW, Sharif SK, Noor AM, Peshu N (2007). The decline in pediatric malaria admissions on the coast of Kenya. Malar J.

[17] O’Meara WP, Bejon P, Mwangi TW, Okiro EA, Peshu N, Snow RW (2008). Effect of a fall in malaria transmission on morbidity and mortality in Kilifi, Kenya. Lancet.

[18] Snow RW, Marsh K (2010). Malaria in Africa: progress and prospects in the decade since the Abuja Declaration. Lancet.

[19] Pathania V (2014). The impact of malaria control on infant mortality in Kenya. Econ Dev Cult Chang.

[20] World Vision US Newsletter (2012). Kenya tops list of 10 countries where infant deaths drop.

[21] Moultrie TA, Dorrington RE, Hill AG, Hill K, Timaeus IM, Zaba B (2013). Tools for Demographic Estimation.

[22] Lawless JF (1982). Statistical Models and Methods for Lifetime Data. Wiley series in probability and mathematical statistics, University of Waterloo, USA.

[23] K’Oyugi BO (2014). Access to child health care, medical treatment of sick children and childhood mortality relationships in Kenya. Health.

[24] Mosley WH, Chen LC (1984). An analytical framework for the study of child survival in developing countries. Popul Dev Rev.

